# Distinct Signature of Oxylipid Mediators of Inflammation during Infection and Asymptomatic Colonization by *E. coli* in the Urinary Bladder

**DOI:** 10.1155/2017/4207928

**Published:** 2017-12-27

**Authors:** Nandakumar Packiriswamy, Jeff Gandy, Sara N. Smith, Harry L. T. Mobley, Lorraine M. Sordillo, Sargurunathan Subashchandrabose

**Affiliations:** ^1^Department of Large Animal Clinical Sciences, Michigan State University, East Lansing, MI, USA; ^2^Department of Microbiology and Immunology, University of Michigan Medical School, Ann Arbor, MI, USA; ^3^Department of Microbiology and Immunology, Wake Forest School of Medicine, Winston-Salem, NC, USA

## Abstract

Urinary tract infection (UTI) is an extremely common infectious disease. Uropathogenic *Escherichia coli* (UPEC) is the predominant etiological agent of UTI. Asymptomatic bacteriuric *E. coli* (ABEC) strains successfully colonize the urinary tract resulting in asymptomatic bacteriuria (ABU) and do not induce symptoms associated with UTI. Oxylipids are key signaling molecules involved in inflammation. Based on the distinct clinical outcomes of *E. coli* colonization, we hypothesized that UPEC triggers the production of predominantly proinflammatory oxylipids and ABEC leads to production of primarily anti-inflammatory or proresolving oxylipids in the urinary tract. We performed quantitative detection of 39 oxylipid mediators with proinflammatory, anti-inflammatory, and proresolving properties, during UTI and ABU caused by genetically distinct *E. coli* strains in the murine urinary bladder. Our results reveal that infection with UPEC causes an increased accumulation of proinflammatory oxylipids as early as 6 h postinoculation, compared to controls. To the contrary, ABEC colonization leads to decreased accumulation of proinflammatory oxylipids at the early time point compared to UPEC infection but does not affect the level of proresolving oxylipids. This report represents the first comprehensive investigation on the oxylipidome during benign ABEC colonization observed in ABU and acute inflammation triggered by UPEC leading to UTI.

## 1. Introduction

Urinary tract infection (UTI) is a ubiquitous infectious condition estimated to affect approximately 150 million people annually in both the developed and the developing world [1]. Women, children, and the elderly are highly predisposed to developing UTI [2]. Antibiotics have remained the primary clinical management strategy for UTI, since their advent and use in the mid-1900s. Indeed, UTI is the second most common reason for antibiotic use in humans [3]. However, rapid global increase in antibiotic resistance in uropathogenic *E. coli* (UPEC) and other uropathogens is a harbinger of the future where UTI might not be resolved with existing antibiotics alone [4]. In the US, 11–21% and 1–6% of UPEC are resistant to trimethoprim-sulfamethoxazole and ciprofloxacin, antimicrobial agents widely used in the treatment of acute cystitis, respectively [5]. These drugs are not used when local resistance rates reach 20% and 10%, respectively [4]. More troubling is the emergence of multidrug-resistant UPEC, including resistance to colistin, which is the antibiotic of last resort against Gram-negative bacteria [6, 7]. Therefore, there is an immediate need to develop novel treatment strategies that function either independently or in synergy with antibiotics to manage UTI.

UPEC is the predominant cause (~85%) of UTI in otherwise healthy individuals [3, 8]. *E. coli* colonization in the urinary tract could lead to completely opposite outcomes of UTI or asymptomatic bacteriuria (ABU) [9]. ABU results from colonization of the urinary tract by *E. coli* strains that are phylogenetically related to UPEC but lack several key virulence factors, including type 1 fimbria and P fimbria that are implicated in the pathogenesis of UTI caused by UPEC [10, 11]. We refer to *E. coli* that induce ABU as asymptomatic bacteriuric *E. coli* (ABEC) in this manuscript. UPEC colonization results in a strong proinflammatory response that is associated with a massive influx of neutrophils and classic symptoms of UTI including dysuria (pain during urination), hematuria (blood in urine), and possibly fever [8]. On the contrary, ABEC colonization results in bacteriuria without symptoms of UTI [12]. Many of the symptoms experienced during UTI reflect the underlying inflammatory response triggered in the host [13]. Notwithstanding the high degree of conservation at the genome level, sparing some virulence factors [14, 15], UPEC and ABEC exhibit disparate phenotypes upon invading the urinary tract.

Oxylipids such as leukotrienes, prostaglandins, and thromboxanes are metabolites derived from fatty acids that play critical roles in shaping the inflammatory response [16]. Understanding the molecular basis of difference in the induction of inflammation by these *E. coli* strains within the urinary bladder has the potential to direct rational design of new strategies to manage UTI. As a first step towards that goal, we investigated the changes in the profile of oxylipid mediators of inflammation during bladder colonization by UPEC and ABEC. The central hypothesis for this study is that UPEC infection triggers production of predominantly proinflammatory oxylipids in the urinary bladder, compared to the oxylipid profile of asymptomatic colonization by ABEC and uninfected controls in the urinary bladder. By utilizing a widely used mouse model [17] and well-characterized strains of UPEC (CFT073) and ABEC (ABU 83972), we present evidence for the first time that colonization of UPEC and ABEC in the urinary bladder results in a distinct signature of oxylipid mediators of inflammation. Our results demonstrate that UPEC colonization leads to increased accumulation of proinflammatory oxylipids derived from the cyclooxygenase and soluble epoxide hydrolase pathways. ABEC colonization results in diminished abundance of proinflammatory oxylipids, compared to UPEC infection. Levels of anti-inflammatory or proresolving oxylipid mediators are not different during ABEC colonization compared to UPEC infection. Our results elucidate the unique profile of oxylipid mediators of inflammation in the pathogenesis of cystitis and asymptomatic colonization caused by *E. coli*.

## 2. Materials and Methods

### 2.1. Bacterial Strains and Culture Conditions

UPEC strain *E. coli* CFT073 was isolated from a hospitalized patient with pyelonephritis and bacteremia [18]. ABEC strain *E. coli* 83792 was isolated from an individual with asymptomatic bacteriuria [10]. *E. coli* strains were cultured in LB medium (tryptone 10 g/l; yeast extract 5 g/l; NaCl 0.05 g/l). Cultures were incubated at 37°C shaking at 200 rpm for 16 h prior to inoculation. Bacteria were pelleted and then suspended in PBS to an OD_600_ of 4.

### 2.2. Mouse Model of UTI

Mouse inoculation experiments were performed in accordance to the protocols (PRO00007111 and PRO00005052) approved by the Institutional Committee at the University of Michigan Medical School. These protocols are compliant with the guidelines published by the Office of Laboratory Animal Welfare (OLAW) and Association for Assessment and Accreditation of Laboratory Animal Care International (AAALAC). UTI was induced in 5- to 7-week-old adult female CBA/J mice (Envigo) as previously described [19] and as recently reviewed in [17]. Briefly, mice were anesthetized and 10^8^ CFU of inoculum in 50 *μ*l PBS was instilled in the urinary bladder transurethrally. Mice in the control group were inoculated with PBS alone. Urine samples were collected at 6 and 48 h postinoculation prior to euthanasia. Turk's stain was used to visualize and enumerate PMNs in a hemocytometer. Cell counts were calculated per ml urine for comparison between samples. Bladders were nicked to remove residual urine, weighed, homogenized in PBS, and plated using an automated plater (Spiral Biotech). Colony counts were determined using Q-Count (Spiral Biotech) to determine bacterial load (CFU/g tissue or CFU/ml urine). Urine-free bladders were utilized for lipodomic analyses as described below.

### 2.3. Chemokine and Cytokine Quantification

Levels of chemokines and cytokines (RANTES, KC, MCP-1, Lix, IL-1*β*, IL-6, IL-10, IL-17*α*, TNF*α*, IFN*β*, and IFN*γ*) in mouse urine samples infected with UPEC or colonized with ABEC at 6 and 48 h postinoculation were determined using a custom quantitative LEGENDplex multi-analyte flow assay kit (BioLegend), according to manufacturer's instructions, and as recently described by Armbruster et al. [20]. Samples, in duplicate, were analyzed in a FACSCanto (BD Biosciences) system and quantified with LEGENDplex data analysis software v7 (BioLegend). Analyte concentration in pg/ml of urine was determined from the standard curve.

### 2.4. Solid-Phase Extraction of Lipids

Oxylipids and fatty acids were quantified using liquid chromatography and tandem mass spectrometry (LC-MS/MS) as described by Ryman et al. [21, 22]. Immediately after collection, urinary bladders were weighed, flash-frozen, and stored in −80°C. Bladders were pulverized in liquid nitrogen using a Mikro-Dismembrator (Sartorius, Germany) and resuspended in PBS. Sonication was carried out in water-cooled sonicator (Misonix) to lyse the cells. Antioxidant and reducing agent mixture (50% methanol, 25% ethanol, and 25% water with 0.9 mM butylated hydroxy toluene, 0.54 mM EDTA, 3.2 mM triphenylphosphine, and 5.6 mM indomethacin) were added to prevent degradation of preformed oxylipids and to prevent ex vivo peroxidation of lipids [23].

Deuterated internal standard mixture was added to each sample to determine the efficiency of recovery of oxylipids from bladder tissue [23]. Internal standard mixture consisted of 5(S)-hydroxyeicosatetraenoic-d_8_ acid [5(S)-HETE_*d*_8_], 15(S)-hydroxyeicosatetraenoic-d_8_ acid [15(S)-HETE_*d*_8_], 8(9)-epoxyeicosatrienoic-d_11_ acid [8(9)-EET_*d*_11_], prostaglandin E2-d_9_ (PGE2_*d*_9_), 8,9-dihydroxyeicosatrienoic-d_11_ acid (8,9-DHET_*d*_11_), and arachidonic acid-d_8_ (AA_*d*_8_), at a final concentration of 0.25, 0.25, 0.5, 0.5, 0.25, and 50 *μ*M, respectively. Recovery of internal standards was used to normalize variation in recovery rate for experimental samples. Unlabeled fatty acid and oxylipid standards were used to generate a 6-point standard curve from 0.001 to 500 *μ*M and 0.01 to 100 nM, respectively.

Methanol was added to all samples at a final concentration of 60%, flash-frozen in liquid nitrogen, and stored at −80°C until analyses. Samples were thawed on ice and centrifuged at 4816 *×*g for 30 min at 4°C with brakes in “off” mode. Methanol concentration was adjusted to 5% with HPLC-grade water. Solid-phase extraction was performed for each urinary bladder sample with Oasis HLB 12 cc (500 mg) LP Extraction Columns (Waters). Columns were conditioned with 6 ml of methanol followed by 6 ml of HPLC-grade water. Extracts were loaded onto the columns, which were then washed with 6 ml of 5% methanol, and the columns were dried for 15 min under full vacuum. Analytes were eluted with 6 ml of methanol:acetonitrile 50 : 50 (vol:vol). The volatile solvents were removed under vacuum using a Savant SpeedVac (ThermoQuest). The residues were reconstituted in 150 *μ*l of methanol:water in 2 : 1 (vol:vol) ratio and transferred to a microcentrifuge tube. The mixture was centrifuged at 14,000 ×g for 10 min at 4°C, and the supernatant was transferred to an autosampler vial with a low volume insert and stored at −80°C until further analysis.

### 2.5. LC-MS/MS Analyses

Quantification of oxylipid metabolites was carried out as described previously [21, 22], using a Waters Acquity UPLC coupled to a Waters Xevo TQ-S triple quadrupole mass spectrometer (Waters) with multiple reaction monitoring. For all tested oxylipids, mentioned above, standard curves were generated to enable precise measurement in mouse urinary bladder samples. Chromatographic separation was performed with an Ascentis Express C18 HPLC column, 10 cm × 2.1 mm, 2.7 *μ*m (Supelco) held at 50°C. Samples were held at 10°C, and the flow rate of the mobile phase was set at 0.3 ml/min. Mobile phase (bottle A) was water with 0.1% formic acid, and mobile phase (bottle B) was acetonitrile. Liquid chromatography separation took 15 min with linear gradient steps programmed as follows (A:B ratio): time 0 to 0.5 min at (99 : 1), to 2.0 min at (60 : 40), to 8.0 min at (20 : 80), to 9.0 min at (1 : 99), 0.5 min held at (1 : 99) until min 13.0, then return to 13.01 min at (99 : 1), and held at this condition until 15.0 min. All oxylipids and fatty acids were detected using electrospray ionization in the negative-ion mode. Cone voltages and collision voltages were optimized for each analyte using Waters QuanOptimize software and multiple reaction monitoring (MRM) parameters were set up as described previously [21, 22]. Data were analysed using MassLynx v4.1 software (Waters).

### 2.6. Abbreviations Used for Targeted Analytes

The abbreviations used for targeted analytes are as follows: PG, prostaglandin; HHTrE, hydroxy heptadecatrienoic acid; TX, thromboxane; EET, epoxy eicosatrienoic acid; DHET, dihydroxy eicosatrienoic acid; HETE, hydroxy eicosatetraenoic acid; DiHETE, dihydroxy eicosatetraenoic acid; EpOME, epoxy octadecenoic acid; DiHOME, dihydroxy octadecenoic acid; EPDPE, epoxy docosapentaenoic acid; DiHDPA, dihydroxy docosapentaenoic acid; HODE, hydroxy octadecadienoic acid; RvD2, resolvin D2; HDoHE, hydroxy docosahexaenoic acid; DiHDoHE, dihydroxy docosahexaenoic acid; HETrE, hydroxyl eicosatrienoic acid; HOTrE, hydroxy octadecatrienoic acid; and HEPE, hydroxy eicosapentaenoic acid.

### 2.7. Statistical Analyses

Data were analyzed by appropriate parametric or nonparametric tests as indicated throughout the manuscript. Statistical analyses were performed using GraphPad Prism Software v7 (San Diego, CA). *P* < 0.05 was considered as a statistically significant difference.

## 3. Results

### 3.1. UPEC and ABEC Exhibit Comparable Potential to Colonize the Mouse Urinary Bladder

**Figure 1:**
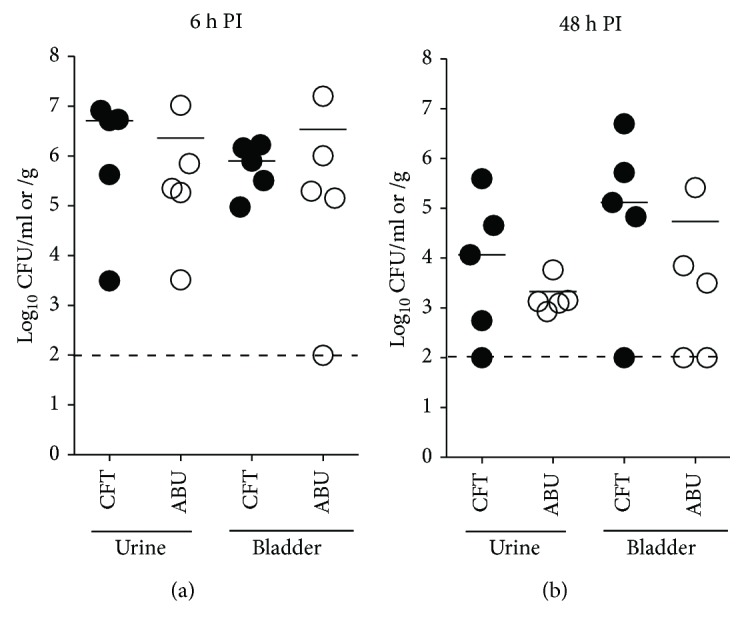
Uropathogenic and asymptomatic bacteriuric *E. coli* colonize murine urinary bladders. Urine and bladder bacterial load in adult, female CBA/J mice (*N* = 5/group/time point) inoculated with 10^8^ CFU of UPEC (CFT, closed circles) or ABEC (ABU, open circles) at 6 h ((a), early acute) and 48 h ((b), late acute) PI. Each symbol represents bacterial load in a mouse, and bars correspond to median CFU. Dotted line denotes the limit of detection (100 CFU). CFT, uropathogenic *E. coli* strain CFT073; ABU, asymptomatic bacteriuria *E. coli* strain 83972; h PI, hour postinoculation.

Female CBA/J mice (*N* = 5/group/time point) were inoculated with 10^8^ CFU of UPEC strain CFT073 or ABEC strain 83972. Urine samples, collected just prior to euthanasia, and bladder homogenates were cultured on LB agar plates to enumerate bacterial load. Both UPEC and ABEC were found in urine and the urinary bladder at the early (6 h, Figure 1(a)) and late (48 h, Figure 1(b)) stages of acute UTI (UPEC) and colonization (ABEC) in the murine model. Median CFU levels were not significantly different between UPEC and ABEC indicating that these strains exhibit comparable competency in colonizing murine urinary tract at the early and late stages of acute infection and colonization, respectively (Figures 1(a) and 1(b)). Compared to 6 h postinoculation, bacterial load of UPEC and ABEC in urine and bladder samples was 1-2 orders of magnitude lower at 48 h postinoculation (Figures 1(a) and 1(b)). Despite completely disparate clinical outcomes in humans, these two *E. coli* strains colonized murine urinary bladder at comparable levels during experimental inoculation.

### 3.2. Host Response during Experimentally Induced UTI and ABU in Mice

**Figure 2:**
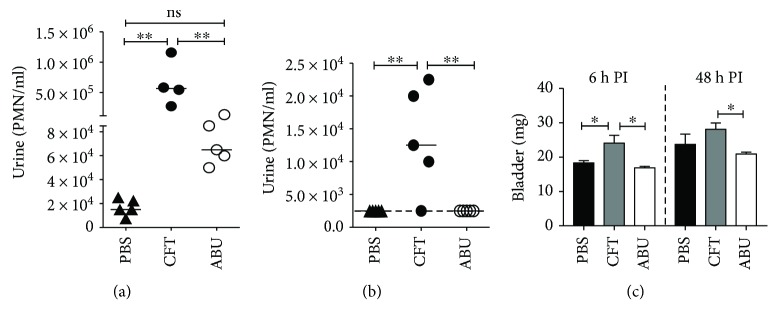
Uropathogenic *E. coli* infection and asymptomatic bacteriuric *E. coli* colonization in mice used for lipidomic analyses. (a, b) Female mice (*N* = 5/group/time point) were inoculated with UPEC (CFT, closed circles) or ABEC (ABU, open circles), and urinary PMN cell count was determined at 6 h (early acute) and 48 h (late acute) postinoculation by Turk's staining. PMNs were counted with a hemacytometer. Each symbol represents PMN count in a mouse, and bars correspond to median. Dotted line denotes the limit of detection (2500 PMN/ml). ^∗∗^*P* < 0.01, Kruskal-Wallis test with Dunn's posttest. (c) Weight of urinary bladders (urine-free) from UPEC-infected and ABEC-colonized mice was determined at 6 h and 48 h PI. These bladders were used for lipidomic analyses. Bars represent mean, and error bars correspond to SEM. ^∗^*P* < 0.05, ANOVA with Dunnett's posttest. PBS, phosphate-buffered saline-inoculated control; CFT, uropathogenic *E. coli* strain CFT073; ABU, asymptomatic bacteriuria *E. coli* strain 83972; PMN, polymorphonuclear cell; and h PI, hour postinoculation.

To collect urinary bladders for targeted lipidomic analyses, mice (female CBA/J, *N* = 5/group) were infected with UPEC strain CFT073 or ABEC strain 83972. Mice in the control group were sham inoculated with PBS. Urine samples were collected at 6 and 48 h postinoculation and used for determining bacterial load, PMN (polymorphonuclear) cell count, and chemokine and cytokine levels. These assays were performed to ensure that the bladders used for lipidomic analysis were indeed exposed to UPEC and ABEC triggering expected host response to infection and colonization, respectively. Urine cultures revealed bacteriuria in mice inoculated with UPEC and ABEC at both early and late time points (similar to Figure 1, data not shown). As expected, urine culture from PBS-inoculated controls did not yield any bacterial colonies. Urinary PMN cell load was determined to measure the degree of neutrophil mobilization response triggered in response to UPEC and ABEC. As predicted, infection with UPEC resulted in a significant increase in the number of urinary PMN cells compared to PBS control and also to ABEC (Figures 2(a) and 2(b)). This is not surprising because ABEC are known to cause asymptomatic colonization in humans. At 48 h postinoculation (Figure 2(b)), urine PMN levels were below the limit of detection (2500 PMN/ml) in the PBS control and ABEC groups compared to UPEC infected mice (median = 12,500 PMN/ml). Higher level of PMN in the PBS controls at 6 h compared to 48 h is due to the sham inoculation procedure. Weight of urinary bladder is an indicator of the degree of tissue inflammation. During the early stage of acute UTI (6 h postinfection), we observed that the bladders from mice infected with UPEC weighed significantly more than the bladders from the PBS and ABEC groups (Figure 2(c)). At 48 h postinfection, bladder weights from all three groups were higher than 6 h time point, indicating a host response to catheter use during inoculation (Figure 2(c)). However, ABEC-inoculated bladders were significantly less heavy than the control and UPEC groups.

**Figure 3:**
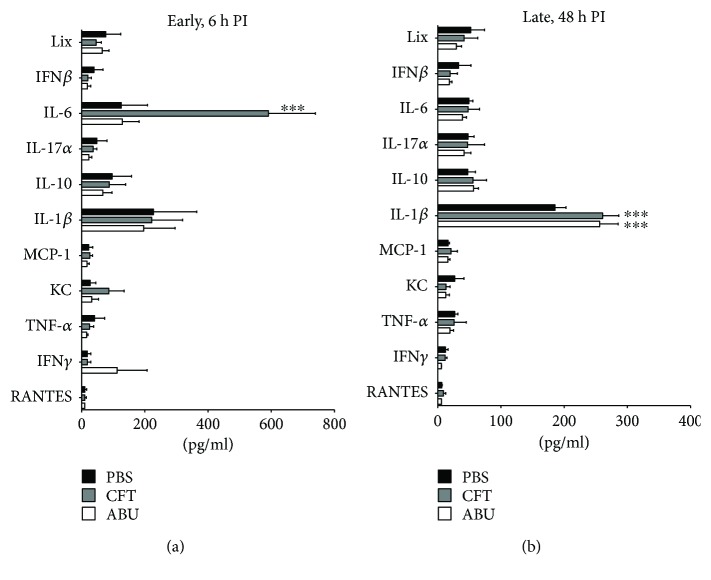
Chemokine and cytokine secretion induced by uropathogenic and asymptomatic bacteriuric *E. coli* in mice. Levels of key chemokines and cytokines in mouse urine (*N* = 5/group/time point) were determined at 6 h (a) and 48 h (b) PI by a quantitative flow cytometry-based method, as described in the Materials and Methods. Bars represent mean, and error bars correspond to SEM. ^∗∗∗^*P* < 0.001, ANOVA with Tukey's multiple comparison test, compared to PBS. PBS, sham-inoculated control; CFT, uropathogenic strain CFT073; ABU, asymptomatic bacteriuric strain 83972; h PI, hour postinoculation.

Next, we tested whether UTI with UPEC and colonization with ABEC induce distinct chemokine and cytokine responses in mice. We measured chemokines and cytokines (Figures 3(a) and 3(b)) that were previously reported to be induced during experimental UTI in the mice [20, 24]. Urinary IL-6 level was significantly higher at 6 h in UPEC-infected mice, compared to the PBS and ABEC groups (Figure 3(a)). There was a trend towards higher level of interferon-*γ* in ABEC-colonized mice at 6 h postinoculation, albeit not statistically significant (Figure 3(a)). At 48 h postinoculation, IL1-*β* levels were higher in both UPEC-infected and ABEC-colonized mice, compared to PBS controls (Figure 3(b)). Taken together, these assays reveal that mice infected with UPEC and colonized with ABEC exhibit different levels of innate immune response as reflected by PMN influx and urinary chemokine and cytokine levels.

### 3.3. Fatty Acid Content of the Urinary Bladder during UTI and ABU

**Figure 4:**
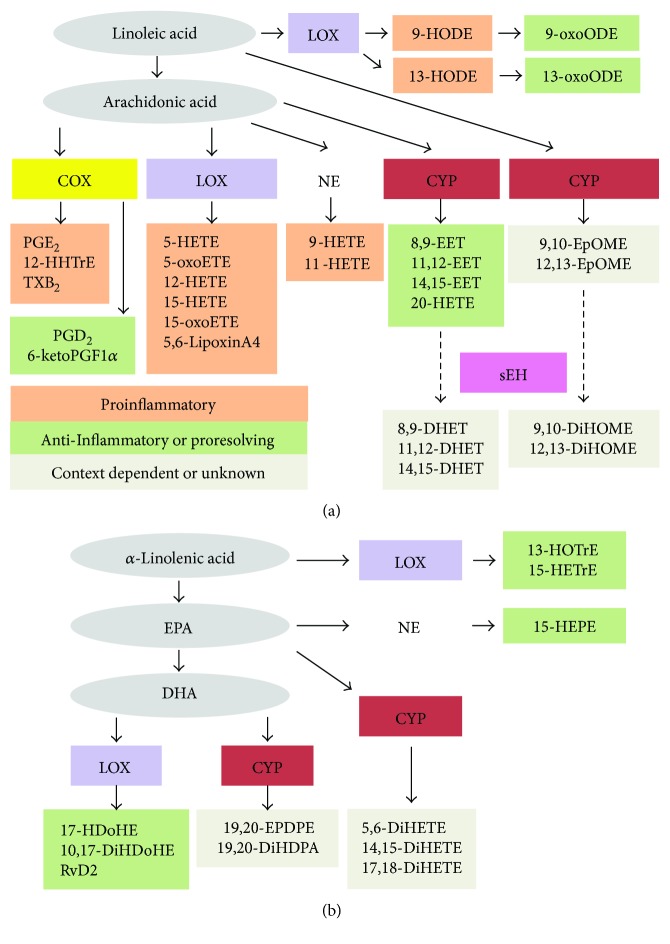
Oxylipid metabolites involved in promoting and resolving inflammation that were probed in this study. This schematic depicts oxylipids derived from linoleic acid (a) and *α*-linolenic acid (b) that were determined in this study. DHA, docosahexaenoic acid; EPA, eicosapentaenoic acid; COX, cyclooxygenase; LOX, lipoxygenase; CYP, cytochrome P450 epoxygenase; sEH, soluble epoxide hydrolase; and NE, nonenzymatic. Chemical names of oxylipids are listed in the Materials and Methods.

**Figure 5:**
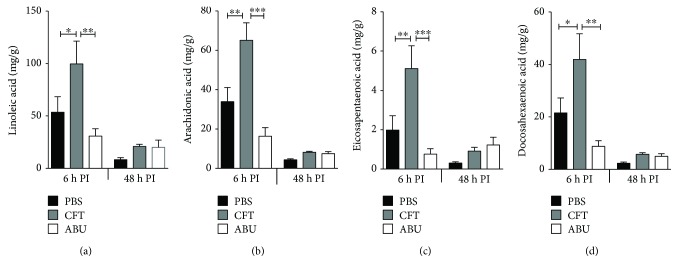
Fatty acid content of the urinary bladder is increased during acute UTI. Fatty acid content of urinary bladders collected from UPEC-infected and ABEC-colonized mice (female CBA/J, *N* = 5/group/time point) was determined by LC-MS/MS. Infection with UPEC strain CFT073 results in significantly higher levels of linoleic acid (a), arachidonic acid (b), eicosapentaenoic acid (c), and docosahexaenoic acid (d) in the bladder compared to PBS controls and ABEC-colonized mice during the onset of UTI (6 h PI). Bladder fatty acid levels at 48 h PI are not significantly different between groups. Mean + SEM are presented here. ^∗^*P* < 0.05, ^∗∗^*P* < 0.01, and ^∗∗∗^*P* < 0.001, ANOVA with Bonferroni's multiple comparison test. PBS, sham-inoculated control; UPEC, uropathogenic *E. coli*; ABEC, asymptomatic bacteriuric *E. coli*; CFT, UPEC strain CFT073; ABU, ABEC strain 83972; h PI, hour postinoculation.

First, we tested whether UPEC and ABEC inoculation results in changes in the concentration of fatty acid precursors of oxylipids in the urinary bladder. Linoleic acid (LA), arachidonic acid (AA), eicosapentaenoic acid (EPA), and docosahexaenoic acid (DHA) are the substrates from which oxylipids are generated during inflammation. Fatty acid substrates and their oxylipid metabolites that were measured in this study are depicted in Figure 4. We determined the concentration of these fatty acids in the urinary bladders of control, UPEC-infected, and ABEC-colonized mice by quantitative liquid chromatography coupled with tandem mass spectrometry (LC-MS/MS) (Figure 5). During the early stage of acute infection (6 h), concentrations of LA, AA, DHA, and EHA were increased in the urinary bladders infected with UPEC, compared to controls (Figures 5(a)–5(d)). ABEC colonization also resulted in a modest decrease in the accumulation of fatty acids at the early stage of colonization, compared to PBS controls (Figure 5). However, this difference was not statistically significant. Compared to acute UTI, ABEC colonization led to significantly lower concentration of LA, AA, EPA, and DHA during the early stage (6 h, Figures 5(b)–5(d)). At the late stage of acute infection and colonization (48 h), the concentrations of fatty acids in the bladders were lower, when compared to 6 h time point (Figures 5(a)–5(d)). Also at 48 h, there was no significant difference in the bladder fatty acid content between the three groups. Next, we profiled the oxylipids derived from these fatty acid substrates in the urinary bladder.

### 3.4. Oxylipid Profiling

**Figure 6:**
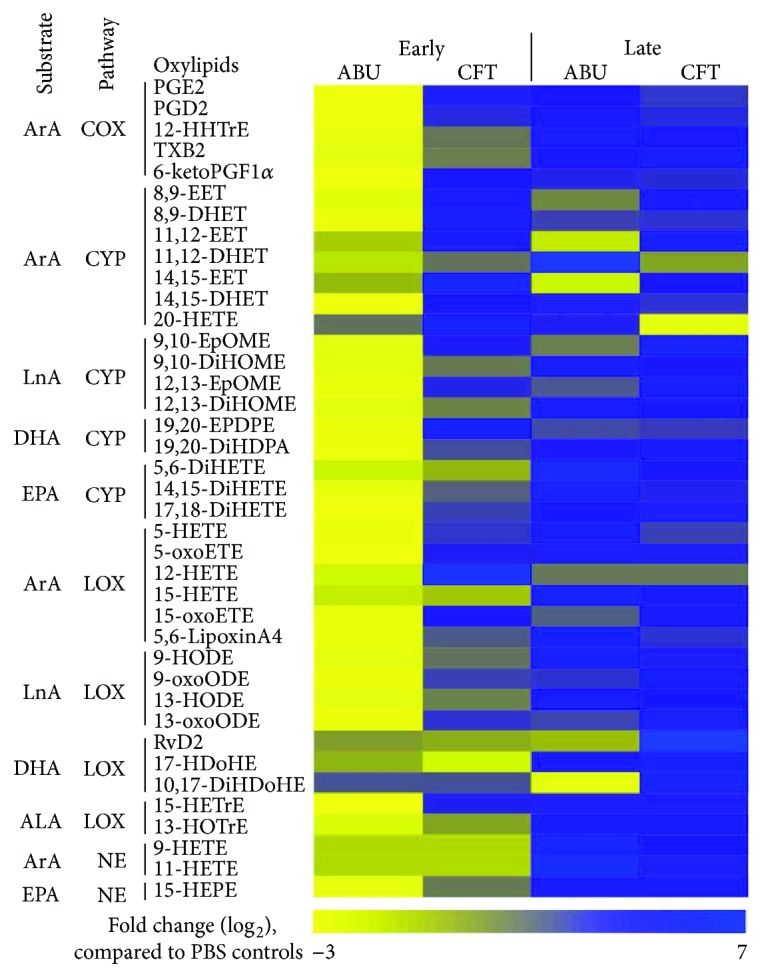
Oxylipid profile of urinary bladders during colonization and infection by *E. coli.* Heatmap depicting fold change in the concentration of 39 oxylipid metabolites in mouse urinary bladders during asymptomatic bacteriuria (ABU) and infection (CFT) by *E. coli*, compared to PBS-inoculated controls, was generated using HeatmapGenerator5. Fold change in abundance compared to PBS controls was calculated and log transformed to generate this heatmap. Substrate and pathways involved in the generation of oxylipid metabolites are also indicated. Adult female CBA/J mice (*N* = 5/group/time point) were used for the early (6 h postinoculation) and late (48 h postinoculation) time points. ABU, asymptomatic bacteriuric *E. coli* strain 83972; CFT, uropathogenic *E. coli* strain CFT073; ArA, arachidonic acid; LnA, linoleic acid; DHA, docosahexaenoic acid; EPA, eicosapentaenoic acid; ALA, *α*-linolenic acid; COX, cyclooxygenase; CYP, cytochrome P450 epoxygenase; sEH, soluble epoxide hydrolase; LOX, lipoxygenase; and NE, nonenzymatic reaction. For analytes that were below the limit of detection (6 h: 9-HETE and 11-HETE; and 48 h: 12-HETE, 15-HETrE, 13-HOTrE, and 15-HEPE), fold change was set at 0. Chemical names of oxylipids are listed in the Materials and Methods.

Oxylipids were extracted from the bladders by solid-phase extraction and quantified using LC-MS/MS. Samples were eluted using a 15 min reverse-phase LC gradient capable of separating most of the known oxylipids as described in [21, 22]. Following LC separation, each oxylipid was selected for identification using multiple reaction monitoring, where the mass spectrometer identified a given molecule by its parent mass and a characteristic fragment ion generated in the second quadrupole. In total, 39 unique oxylipid metabolites derived from cyclooxygenase (COX), lipoxygenase (LOX), cytochrome P450 epoxygenase (CYP), soluble epoxide hydrolase (sEH), and nonenzymatic (NE) pathways were quantified in the urinary bladder at the early (6 h) and late (48 h) stages after experimental inoculation of UPEC and ABEC (Table 1). Global view of the comparative bladder oxylipidome depicts markedly different levels of accumulation of various oxylipids during *E. coli* infection compared to benign colonization by ABEC (Figure 6). UPEC infection led to increased accumulation of several proinflammatory oxylipids at both the early and late stages of acute infection, compared to the ABEC and PBS control groups (Table 1). This increase is also reflected in an increased fold change in oxylipid content during UTI, compared to ABU and uninfected controls (Figure 6). In contrast, ABEC colonization led to a considerable decrease in proinflammatory oxylipids at the early stage (6 h), followed by a late surge in proinflammatory oxylipid production at 48 h postinoculation, as compared to PBS controls (Figure 6).

### 3.5. Inflammatory Mediators Produced from Cyclooxygenase Pathway during UTI and ABU

**Figure 7:**
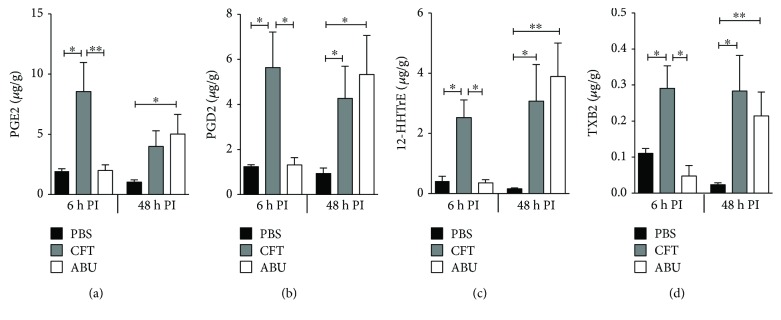
Oxylipids derived from cyclooxygenase activity are found at higher levels during *E. coli* infection and colonization. LC-MS/MS was used to determine the levels of prostaglandins E2 (a) and D2 (b), 12-hydroxy heptadecatrienoic acid (c), and thromboxane B2 (d) in mouse urinary bladders (female CBA/J, *N* = 5/group/time point). Mean + SEM are presented here. ^∗^*P* < 0.05 and ^∗∗^*P* < 0.01, ANOVA with Bonferroni's multiple comparison test. PBS, sham-inoculated control; UPEC, uropathogenic *E. coli*; ABEC, asymptomatic bacteriuric *E. coli*; CFT, UPEC strain CFT073; ABU, ABEC strain 83972; h PI, hour postinoculation.

Arachidonic acid is released from the cytoplasmic membrane by the action of phospholipase A2 [16]. Cyclooxygenases (COX) catalyze the production of prostaglandins and thromboxanes from arachidonic acid as depicted in Figure 4 [16]. We found a significantly increased concentration of proinflammatory oxylipids prostaglandin E2 (PGE2), 12-hydroxy heptadecatrienoic acid (HHTrE), and thromboxane B2 (TXB2) in the mouse bladders during acute UTI, as compared to the ABU and PBS control groups (Figures 7(a), 7(c), and 7(d)). Prostaglandin D2 (PGD2) is an anti-inflammatory oxylipid whose content was also increased during acute UTI, compared to controls (Figure 7(b)). However, at the late stage of acute UTI (48 h), concentrations of PGD2, 12-HHTrE, and TXB2 were significantly increased in both the UPEC and ABEC groups, compared to PBS controls (Figures 7(b)–7(d)). Interestingly, concentration of these oxylipids was not significantly different between the UPEC and ABEC groups at 48 h (Figure 7). PGE2 concentration revealed a trend towards increase, although not statistically significant, at 48 h in UPEC- and ABEC-inoculated mice (Figure 7(a)). The concentration of PGE2, PGD2, 12-HHTrE, and TXB2 in mice with ABU at early stage was significantly lower than that of the UTI group and comparable to that of PBS controls (Figures 7(a)–7(d)). However, there was a significant increase in the accumulation of PGE2, PGD2, 12-HHTrE, and TXB2 during the late stage (48 h) of colonization by ABEC (Figures 7(a)–7(d)).

### 3.6. Oxylipids Synthesized by Cytochrome P450 Epoxygenase in the Urinary Bladder

**Figure 8:**
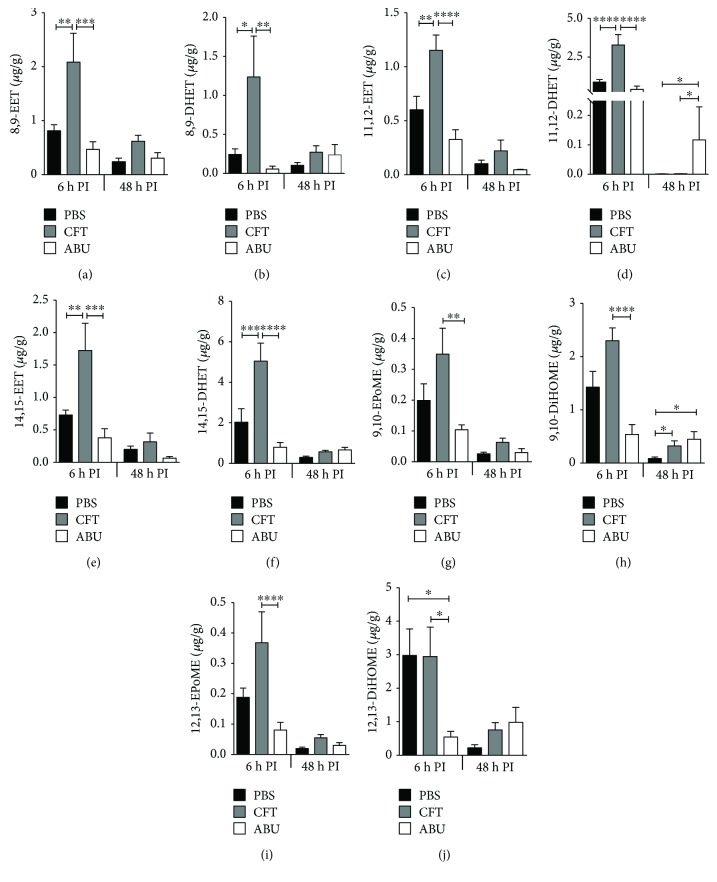
UPEC infection and ABEC colonization induce production of oxylipids from the cytochrome P450 epoxygenase pathway. Levels of 8,9-EET, 8,9-DHET, 11,12-EET, 11,12-DHET, 14,15-EET, 14,15-DHET, 9,10-EPOME, 9,10-DiHOME, 12,13-EPOME, and 12,13-DiHOME in mouse urinary bladders inoculated with PBS, UPEC, and ABEC were determined by LC-MS/MS (female CBA/J, *N* = 5/group/time point). Mean + SEM are presented here. ^∗^*P* < 0.05, ^∗∗^*P* < 0.01, ^∗∗∗^*P* < 0.001, and ^∗∗∗∗^*P* < 0.001, ANOVA with Bonferroni's multiple comparison test. PBS, sham-inoculated control; UPEC, uropathogenic *E. coli*; ABEC, asymptomatic bacteriuric *E. coli*; CFT, UPEC strain CFT073; ABU, ABEC strain 83972; h PI, hour postinoculation; EET, epoxy eicosatrienoic acid; DHET, dihydroxy eicosatrienoic acid; EpOME, epoxy octadecenoic acid; and DiHOME, dihydroxy octadecenoic acid.

Cytochrome P450 epoxygenase (CYP) catalyzes the production of hydroxy eicosatetraenoic acids (HETEs), epoxy eicosatrienoic acids (EETs), and epoxides from linoleic acid, arachidonic acid, eicosapentaenoic acid, and docosahexaenoic acid [16], as shown in Figure 4. In this study, we quantified 16 oxylipids generated by the CYP pathway in urinary bladders infected with UPEC and colonized by ABEC. We found that the concentration of archidonic acid-derived EETs (8,9-EET, 11,12-EET, and 14,15-EET) and their less active downstream metabolic products DHETs (dihydroxy eicosatrienoic acids: 8,9-DHET, 11,12-DHET, and 14,15-DHET) were significantly increased in the mouse urinary bladders during the early stage of acute UTI, compared to ABU and PBS controls (Figures 8(a)–8(f)). At 48 h postinoculation, the concentrations EETs and DHETs in the UTI group were comparable to the ABU and PBS control groups. Compared to the early stage of acute UTI, concentrations of EETs and DHETs were significantly lower during the late stage (Figures 8(a)–8(f)). However, there is no marked change in EET and DHET concentrations during the early and late phases of ABEC colonization. In comparison, concentrations of linoleic acid-derived CYP pathway metabolites (9,10-EpOME and 12,13-EpOME) and their respective less active metabolites (9,10-DiHOME and 12,13-DiHOME) were higher during UTI, compared to the ABU group at 6 h (Figures 8(g)–8(j)). Based on the concentration of EPoME and DiHOME in the urinary bladders of PBS control mice at 6 h time point, it appears that ABEC inoculation suppresses the activation of this pathway.

### 3.7. Oxylipids Generated from Lipoxygenase Pathway in the Urinary Bladder during UTI and ABU

**Figure 9:**
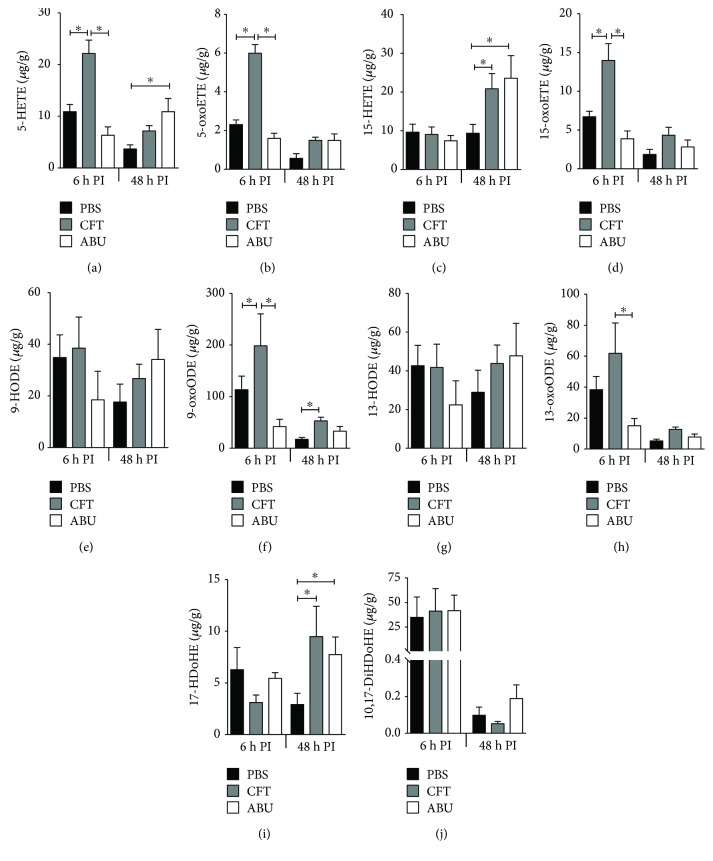
LOX pathway-derived oxylipids induced during infection and asymptomatic colonization of the murine urinary tract. Oxylipids in murine urinary bladders were quantified by LC-MS/MS (female CBA/J, *N* = 5/group/time point). Mean + SEM are presented here. ^∗^*P* < 0.05, ANOVA with Bonferroni's multiple comparison test. PBS, sham-inoculated control; UPEC, uropathogenic *E. coli*; ABEC, asymptomatic bacteriuric *E. coli*; CFT, UPEC strain CFT073; ABU, ABEC strain 83972; h PI, hour postinoculation; HETE, hydroxy eicosatetraenoic acid; HODE, hydroxy octadecadienoic acid; HDoHE, hydroxy docosahexaenoic acid; and DiHDoHE, dihydroxy docosahexaenoic acid.

Lipoxygenases catalyze the conversion of polyunsaturated fatty acids into conjugated hydroperoxides [16]. We determined 15 LOX-dependent oxylipid molecules of which 8 were proinflammatory and 7 anti-inflammatory/proresolving. Concentrations of arachidonic acid metabolites (5-HETE, 5-oxoETE, and 15-oxoETE) were significantly higher in the UTI group compared to both the PBS and ABEC groups at 6 h postinoculation (Figure 9). At 48 h postinoculation, the concentrations of 9-oxoODE and 17-HDoHE were lower in mice with UTI compared to the PBS group (Figure 9). Additionally, concentration of 5-HETE was lower in the UPEC group in comparison to both PBS and ABEC groups at 48 h postinoculation (Figure 9). Both UTI and ABU resulted in increased accumulation of 15-HETE and 17-HDoHE in the bladders at the late time point.

Concentration of docosahexaenoic acid- (DHA-) derived metabolites was also altered during UTI. Specifically, at 6 h postinoculation, concentration of 17-HDoHE was decreased in the CFT group compared to the ABEC group. At 48 h postinoculation, 17-HDoHE concentration was increased in the ABEC and CFT groups compared to the PBS group (Figure 9). Similarly, concentration of 10,17-DiHDoHE (protectin D1), a proresolving oxylipid and a downstream metabolite of 17-HDoHE, was decreased in the CFT group 48 h postinoculation compared to the ABEC group (Figure 9). In summary, both pro- and anti-inflammatory lipoxygenase-dependent oxylipid molecules were generated in the bladder during UTI.

## 4. Discussion

Inflammation of the urinary bladder (cystitis) is a defining feature of the most common form of clinical UTI and leads to the manifestation of symptoms observed during UTI [8]. Although the inflammatory response is triggered to clear pathogens, collateral damage that is inflicted upon the host tissue is well documented [25]. Therefore, understanding the role of inflammatory mediators in *E. coli* colonization and infection in the urinary bladder has the potential to open up new clinical management strategies against UTI. Oxylipids are a family of potent lipid mediators that regulate the onset, magnitude, and resolution of microbial infections [26]. Emerging evidence suggests that oxylipid generation is more complex than previously appreciated and newly identified oxylipids are ascribed novel roles in modulating inflammation [16]. Mouse model of UTI has been used for more than three decades to investigate host-pathogen interaction and ensuing inflammation in the urinary tract [9, 17, 27, 28]. However, the oxylipidome of urinary bladder during infection has not been characterized. In this study, we bridge this important gap in knowledge by exploring the role of oxylipid metabolites of linoleic acid, arachidonic acid, eicosapentaenoic acid, and docosahexaenoic acid in the urinary bladder during *E. coli* colonization and infection. Applying LC-MS/MS technology, here, we report the concentration of 39 oxylipid metabolites, their degradation products, and precursor molecules in the urinary bladder during acute UTI and asymptomatic *E. coli* colonization in the mouse model.

Recent studies have highlighted the involvement of bioactive oxylipid mediators of inflammation in bacterial and viral diseases. Experimental peritonitis with *E. coli* and skin infection with *Staphylococcus aureus* in a mouse model have elucidated the role of oxylipids that promote resolution of inflammation, including resolvin D1, resolvin D5, and protectin D1, as critical effectors of bacterial clearance [29]. Polymorphisms at *LTA4H* locus, leading to increased production of leukotriene B4, were associated with decreased susceptibility to tuberculosis in humans [30]. This clinical observation is supported by mechanistic studies in a zebra fish model of *Mycobacterium marinum* infection where optimal production of leukotriene B4 and concomitant proinflammatory response are essential for protection against *M. marinum* infection [30]. Oxylipidomic profiling of permissive and resistant mouse strains infected with *Borrelia burgdorferi*, the etiological agent of Lyme disease, elucidated key differences in oxylipid concentration that correlated with differences in the degree of development of arthritis [23]. In addition to bacterial infections, viral infections also result in a distinct oxylipid signature in the host. Tam et al. reported the unique profile of oxylipids during influenza virus infection in a mouse model and found that this response is also conserved in samples from patients with flu [31]. Taken together with our report on oxylipids involved in UTI and asymptomatic colonization, these studies highlight that elucidating the oxylipid mediators of inflammation is needed for a comprehensive understanding of the complex crosstalk at the host-pathogen interface.

Utilizing a mouse model of UTI, Hannan and coworkers have reported that early inhibition of cycloxygenase activity decreases the incidence of recurrent UTI [32]. Their proteomic approach revealed elevated expression of cyclooxygenase during the early stages of experimental UTI in mouse urinary bladders. Furthermore, cyclooxygenase induction was primarily found in the neutrophils and macrophages that are recruited to the urinary bladder in response to UPEC inoculation [32]. However, a comprehensive understanding of the identity of oxylipid metabolites involved in this process is lacking. Our study bridges that knowledge gap by directly profiling the bioactive oxylipid molecules found in the bladder during ABU and UTI caused by clinical ABEC and UPEC strains, respectively. Our study reveals a differential neutrophil mobilization and inflammatory response in murine urinary bladder following UPEC infection and ABEC colonization. This difference is of special interest because of the previously known roles of neutrophils in promoting chronic and recurrent UTI in murine models, including by oxylipid biosynthesis from the cyclooxygenase-2 pathway [32]. The overall change in fatty acid accumulation during UTI and ABU is consistent with the downstream effects on oxylipid levels and a concomitant difference in triggering a proinflammatory host response (UPEC) versus the failure to induce inflammation (ABEC). Temporal difference in accumulation of oxylipids between UTI and ABU was an unexpected outcome of this study and suggests that late accumulation of proinflammatory oxylipids might confer protection against severe tissue damage during UTI. Further experiments are required to delineate the specific contribution of temporal difference in oxylipid levels on the outcome of UTI and ABU. Our results unravel the role of soluble epoxide hydrolase in degrading anti-inflammatory oxylipids in the bladder. Further studies are required to determine the relative contribution of bladder epithelial cells, macrophages, and neutrophils in oxylipid biogenesis within the bladder during UTI.

Unwarranted use of antibiotics contributes to the emergence of multi- and extreme drug-resistant bacterial pathogens and also perturbs the normal host microbiome [4]. Currently, UTI is the second leading cause of prescription antibiotic use in the United States [3]. Modulating inflammation to manage UTI represents a novel approach that could minimize development of antibiotic resistance by limiting the use of clinically effective antibiotics for UTI. Medications that control inflammation, such as acetaminophen and ibuprofen, are among the most commonly used drugs globally. These drugs inhibit the action of cyclooxygenase-2 that catalyzes the production of prostaglandins and thromboxanes, key mediators of inflammation, from arachidonic acid [33]. Because of the central role of inflammation in the symptoms exhibited during UTI, clinical trials were conducted to determine the effect of suppressing inflammation with an analgesic during acute uncomplicated UTI in otherwise healthy women, compared to antibiotic treatment [34, 35]. In a pilot clinical trial with 40 UTI patients in the ibuprofen group and 39 UTI patients in the ciprofloxacin group, the authors found no significant difference in the duration of symptoms between the antibiotic and analgesic groups [35]. However, due to the small sample size, that study was not adequately powered to draw a definitive conclusion. Another clinical trial with adequate statistical discriminatory power revealed a significant decrease in antibiotic use in patients with acute uncomplicated UTI, when ibuprofen was prescribed instead of antibiotics at the first visit [34]. Around 66% of UTI patients who received ibuprofen recovered from UTI without administration of antibiotics [34]. However, total symptom burden was higher in the analgesic group, compared to patients who received antibiotics. It must be noted that there was a considerable increase in the cases of pyelonephritis in the analgesic group compared to the antibiotic group. These encouraging results demonstrate a clear need for further research on the criteria to identify suitable patients for treatment with analgesics instead of antibiotics at the first visit. By enhancing the clearance of *E. coli* and *S. aureus*, oxylipids involved in resolution of inflammation (resolvin D1, resolvin D5, and protectin D1) decrease the amount of antibiotics required for complete bacterial killing, compared to controls, in murine models [29]. Taken together with our work in the mouse model of UTI described here and a previous report [32], modulating the bioactive oxylipids involved in inflammation is emerging as a new strategy to manage UTI.

Here, we report that multiple cyclooxygenase-generated proinflammatory metabolites including prostaglandins E2 and D2 and thromboxane B2 are highly abundant during UTI in the mouse urinary bladder. Expression and activity of inducible isozyme cyclooxygenase-2 are regulated by many factors encountered at the milieu of inflammation such as NF-*κ*B, IL-1*β*, lipopolysaccharide, and TNF-*α* [36], which are found in the bladder during UTI [28]. However, complete inhibition of cyclooxygenase activity might be detrimental for the host because cyclooxygenases are critical for optimal host defense during bacterial infection. Mice lacking cyclooxygenase-2 are susceptible to severe UTI marked by increased *E. coli* load in urinary bladders during experimental infection, compared to wild-type mice [37]. UPEC induces cyclooxygenase-2 expression in cells involved in inflammation in the mouse bladder during UTI [38]. Inflammation in the bladder increases the levels and activity of cyclooxygenase in humans. Cyclooxygenase-2 protein and its activity, as measured by the production of prostaglandin E2, were higher in human urine during UTI and also in patients with bladder cancer [39]. UPEC induces the expression and activity of cyclooxygenase-2 in human bladder cells *in vitro* [40]. Specifically, type 1 fimbria produced by UPEC induces cyclooxygenase-2 expression by signaling via TLR-4 in human bladder epithelial cells [40]. On the contrary, ABEC strain 83972 lacking type 1 fimbria does not induce cyclooxygenase-2 expression. This *in vitro* observation in human cells is supported by our *in vivo* finding in mouse bladders on the differential nature of cyclooxygenase-2 induction, as measured by the abundance of prostaglandin E2, prostaglandin D2, and thromboxane B2, by UPEC and ABEC. These differences in oxylipid profile might explain, at least in part, striking differences in the outcome of colonization leading to ABU or UTI.

Our results reveal an increase in prostaglandin E2 level in the murine urinary bladder during UTI. Consistent with our findings, Duell et al. have reported increased abundance of prostaglandin E synthase and prostaglandin-endoperoxide synthase transcripts in murine urinary bladders (C57BL/6 and CBA strains) as early as 2 h postinoculation with UPEC strain CFT073 [41]. Production of prostaglandin E2 in the detrusor muscle is triggered by bacteria-associated signals [42]. Activity of the detrusor muscle is critical for voiding during micturition. Macrophages located within the detrusor muscle produce prostaglandins (E2 and F2*α*) by upregulating the expression of cyclooxygenase-2 when stimulated with formylated peptides, a surrogate for bacterial infection, to increase the activity of detrusor muscle. Indomethacin, an inhibitor of both cyclooxygenase-1 (constitutively expressed) and cyclooxygenase-2 (induced during inflammation), abolishes this effect and links the activity of cyclooxygenases to the function of detrusor muscle [42]. In addition to UTI, targeting oxylipid metabolites could be explored for management of bladder conditions marked by increased detrusor muscle activity including overactive bladder disease and lower urinary tract symptoms.

Soluble epoxide hydrolases catalyze the conversion of more active anti-inflammatory oxylipids, such as 8,9-EET and 11,12-EET, into less active diol derivatives including 8,9-DHET and 11,12-DHET [43]. Inhibitors of soluble epoxide hydrolase hamper inflammation by maintaining high levels of anti-inflammatory oxylipids [43]. By inhibiting the activity of soluble epoxide hydrolase, mice were protected from LPS-induced mortality that is marked by severe inflammation. Importantly, our results highlight the fact that specific pathways can now be targeted to promote resolution of inflammation during UTI rather than completely blocking inflammatory response. Based on the findings of this study, work is in progress to determine the efficacy of blocking soluble epoxide hydrolase on limiting tissue damage and symptoms caused by inflammation during UTI. In summary, we present the first comprehensive oxylipidome of the urinary bladder during infection and asymptomatic colonization by *E. coli*. This study will serve as a framework for future translational investigations on modulating inflammation to restore urinary tract health.

## Figures and Tables

**Table 1:** Oxylipidome of the urinary bladder during *E. coli* colonization and infection.

Substrate: pathway		Early, 6 h PI			Late, 48 h PI		
	Oxylipid	PBS	ABU	UTI-CFT	PBS	ABU	UTI-CFT
ArA:COX	PGE2	1.94 ± 0.2	1.99 ± 0.5	8.55 ± 2.4	1.03 ± 0.2	5.02 ± 1.6	3.99 ± 1.3
ArA:COX	PGD2	1.24 ± 0.08	1.31 ± 0.3	5.64 ± 1.6	0.93 ± 0.2	5.3 ± 1.7	4.27 ± 1.4
ArA:COX	12-HHTrE	0.42 ± 0.1	0.35 ± 0.1	2.53 ± 0.58	0.16 ± 0.03	3.90 ± 1.1	3.08 ± 1.2
ArA:COX	TXB2	0.11 ± 0.01	0.047 ± 0.03	0.29 ± 0.06	0.024 ± 0.01	0.21 ± 0.06	0.28 ± 0.01
ArA:COX	6-ketoPGF1*α*	7.66 ± 2.07	4.4 ± 1.4	42.5 ± 10.6	1.3 ± 0.3	5.55 ± 1.17	5.32 ± 1.6
ArA:CYP	8,9-EET	0.81 ± 0.1	0.46 ± 0.1	2.08 ± 0.53	0.24 ± 0.06	0.30 ± 0.10	0.62 ± 0.1
ArA:CYP-sEH	8,9-DHET	0.24 ± 0.1	0.056 ± 0.04	1.24 ± 0.52	0.10 ± 0.03	0.24 ± 0.1	0.27 ± 0.08
ArA:CYP	11,12-EET	0.60 ± 0.1	0.33 ± 0.01	1.15 ± 0.14	0.10 ± 0.03	0.04 ± 0.01	0.22 ± 0.09
ArA:CYP-sEH	11,12-DHET	0.88 ± 0.16	0.4 ± 0.2	3.3 ± 0.67	0.001 ± 0.003	0.12 ± 0.1	0.002 ± 0.003
ArA:CYP	14,15-EET	0.73 ± 0.1	0.38 ± 0.14	1.73 ± 0.42	0.2 ± 0.05	0.065 ± 0.01	0.32 ± 0.13
ArA:CYP-sEH	14,15-DHET	2.03 ± 0.66	0.79 ± 0.2	5.04 ± 0.89	0.29 ± 0.001	0.66 ± 0.13	0.56 ± 0.07
ArA:CYP	20-HETE	0.047 ± 0.01	0.054 ± 0.01	0.114 ± 0.02	0.30 ± 0.13	0.51 ± 0.15	0.13 ± 0.03
LnA:CYP	9,10-EpOME	0.199 ± 0.1	0.1 ± 0.02	0.349 ± 0.08	0.03 ± 0.001	0.03 ± 0.01	0.06 ± 0.01
LnA:CYP-sEH	9,10-DiHOME	1.43 ± 0.29	0.54 ± 0.19	2.3 ± 0.24	0.09 ± 0.01	0.45 ± 0.14	0.32 ± 0.01
LnA:CYP	12,13-EpOME	0.188 ± 0.03	0.08 ± 0.02	0.368 ± 0.10	0.02 ± 0.005	0.03 ± 0.009	0.05 ± 0.01
LnA:CYP-sEH	12,13-DiHOME	2.98 ± 0.78	0.55 ± 0.16	2.95 ± 0.88	0.22 ± 0.09	0.98 ± 0.45	0.76 ± 0.21
DHA:CYP	19,20-EPDPE	0.96 ± 0.29	0.67 ± 0.13	1.63 ± 0.68	0.84 ± 0.01	1.08 ± 0.040	1.12 ± 0.09
DHA:CYP	19,20-DiHDPA	8.88 ± 2.1	2.44 ± 0.44	16.51 ± 6.53	0.92 ± 0.2	2.60 ± 0.53	3.11 ± 0.32
EPA:CYP	5,6-DiHETE	0.27 ± 0.22	0.25 ± 0.21	0.42 ± 0.1	0.023 ± 0.1	0.19 ± 0.07	0.11 ± 0.04
EPA:CYP	14,15-DiHETE	1.97 ± 1.1	0.57 ± 0.14	2.84 ± 1.4	0.31 ± 0.01	1.15 ± 0.32	0.69 ± 0.20
EPA:CYP	17,18-DiHETE	16.48 ± 5.16	4.18 ± 1.89	25.22 ± 3.98	2.52 ± 0.43	6.19 ± 1.18	5.04 ± 0.79
ArA:LOX	5-HETE	10.9 ± 1.39	6.3 ± 1.6	22.15 ± 2.6	3.68 ± 0.78	10.89 ± 2.56	7.16 ± 1.04
ArA:LOX	5-oxoETE	2.3 ± 0.24	1.59 ± 0.27	5.99 ± 0.44	0.56 ± 0.02	1.49 ± 0.34	1.5 ± 0.17
ArA:LOX	12-HETE	290.6 ± 82.1	192.4 ± 80.1	503.5 ± 95.8	BLD	BLD	BLD
ArA:LOX	15-HETE	9.65 ± 2.1	7.42 ± 1.37	9.04 ± 1.91	9.36 ± 2.29	23.59 ± 5.83	20.86 ± 3.9
ArA:LOX	15-oxoETE	6.72 ± 0.7	3.88 ± 1.0	13.99 ± 2.2	1.85 ± 0.66	2.8 ± 0.9	4.32 ± 1.04
ArA:LOX	5,6-LipoxinA4	0.397 ± 0.1	0.139 ± 0.05	0.75 ± 0.37	0.162 ± 0.06	0.753 ± 0.23	0.403 ± 0.09
LnA:LOX	9-HODE	34.83 ± 8.84	18.48 ± 11.1	38.49 ± 12.0	17.62 ± 6.94	34.1 ± 11.71	26.72 ± 5.54
LnA:LOX	9-oxoODE	113.4 ± 26.2	41.8 ± 14.25	198.3 ± 61.9	17.41 ± 3.48	33.2 ± 8.8	52.9 ± 7.25
LnA:LOX	13-HODE	42.62 ± 10.5	22.41 ± 12.41	41.75 ± 12.1	28.9 ± 11.4	47.7 ± 16.8	43.76 ± 9.5
LnA:LOX	13-oxoODE	38.33 ± 8.63	15.1 ± 4.7	61.87 ± 19.6	5.2 ± 1.2	7.77 ± 1.95	12.7 ± 1.5
DHA:LOX	RvD2	0.053 ± 0.03	0.087 ± 0.03	0.077 ± 0.02	0.044 ± 0.02	0.068 ± 0.02	0.05 ± 0.01
DHA:LOX	17-HDoHE	6.29 ± 2.1	5.45 ± 0.53	3.11 ± 0.72	2.91 ± 1.08	7.75 ± 1.69	9.49 ± 2.93
DHA:LOX	10,17-DiHDoHE	34.82 ± 20.8	41.17 ± 22.9	41.64 ± 15	0.099 ± 0.04	0.05 ± 0.01	0.19 ± 0.07
ALA:LOX	15-HETrE	24.1 ± 7.26	9.93 ± 3.59	23.54 ± 6.6	BLD	BLD	BLD
ALA:LOX	13-HOTrE	180.6 ± 66.42	48.88 ± 24.2	82.62 ± 21.3	BLD	BLD	BLD
ArA:NE	9-HETE	BLD	BLD	BLD	1.59 ± 0.38	2.14 ± 0.63	2.02 ± 0.45
ArA:NE	11-HETE	BLD	BLD	BLD	4.09 ± 1.3	10.6 ± 3.3	7.8 ± 1.6
EPA:NE	15-HEPE	49.35 ± 21.16	19.2 ± 4.6	33.75 ± 6.67	BLD	BLD	BLD

Concentration in *μ*g/g of bladder. h PI: hour postinoculation; PBS: control; ABU: asymptomatic bacteriuric *E. coli* colonization; UTI-CFT: urinary tract infection with uropathogenic *E. coli* strain CFT073; ArA: arachidonic acid; LnA: linoleic acid; DHA: docosahexaenoic acid; EPA: eicosapentaenoic acid; ALA: *α*-linoleic acid; COX: cyclooxygenase; CYP: cytochrome P450 epoxygenase; sEH: soluble epoxide hydrolase; LOX: lipoxygenase; BLD: below limit of detection. Mean and SEM from five mice/group/time point. Chemical names of oxylipids are provided in the Materials and Methods.
